# Re-framing the importance of Group B *Streptococcus* as a gut-resident pathobiont

**DOI:** 10.1128/iai.00478-23

**Published:** 2024-03-04

**Authors:** Joie Ling, Andrew J. Hryckowian

**Affiliations:** 1 Department of Medicine, Division of Gastroenterology and Hepatology, University of Wisconsin School of Medicine and Public Health, Madison, Wisconsin, USA; 2 Department of Medical Microbiology and Immunology, University of Wisconsin School of Medicine and Public Healthon, Madison, Wisconsin, USA; 3 Microbiology Doctoral Training Program, University of Wisconsin-Madison, Madison, Wisconsin, USA; University of Pittsburgh, Pittsburgh, Pennsylvania, USA

**Keywords:** *Streptococcus agalactiae*, gut microbiome, human microbiome, pathogenesis, microbial ecology

## Abstract

*Streptococcus agalactiae* (Group B *Streptococcus*, GBS) is a Gram-positive bacterial species that causes disease in humans across the lifespan. While antibiotics are used to mitigate GBS infections, it is evident that antibiotics disrupt human microbiomes (which can predispose people to other diseases later in life), and antibiotic resistance in GBS is on the rise. Taken together, these unintended negative impacts of antibiotics highlight the need for precision approaches for minimizing GBS disease. One possible approach involves selectively depleting GBS in its commensal niches before it can cause disease at other body sites or be transmitted to at-risk individuals. One understudied commensal niche of GBS is the adult gastrointestinal (GI) tract, which may predispose colonization at other body sites in individuals at risk for GBS disease. However, a better understanding of the host-, microbiome-, and GBS-determined variables that dictate GBS GI carriage is needed before precise GI decolonization approaches can be developed. In this review, we synthesize current knowledge of the diverse body sites occupied by GBS as a pathogen and as a commensal. We summarize key molecular factors GBS utilizes to colonize different host-associated niches to inform future efforts to study GBS in the GI tract. We also discuss other GI commensals that are pathogenic in other body sites to emphasize the broader utility of precise de-colonization approaches for mitigating infections by GBS and other bacterial pathogens. Finally, we highlight how GBS treatments could be improved with a more holistic understanding of GBS enabled by continued GI-focused study.

## GBS AS A PATHOGEN IN HUMANS ACROSS THE LIFESPAN

*Streptococcus agalactiae* (Group B *Streptococcus*, GBS) is a Gram-positive bacterial species that causes disease in humans across the lifespan. Patient populations impacted by GBS include pregnant people, neonates, fetuses, and adults/children with underlying health conditions. The incidence of GBS disease in non-pregnant adults, in particular, rose from 8.1 cases per 100,000 people in 2008 to 10.9 cases per 100,000 people in 2016 in the United States (1), which highlights one important facet of the increasing disease burden imposed by GBS.

**Fig 1:**
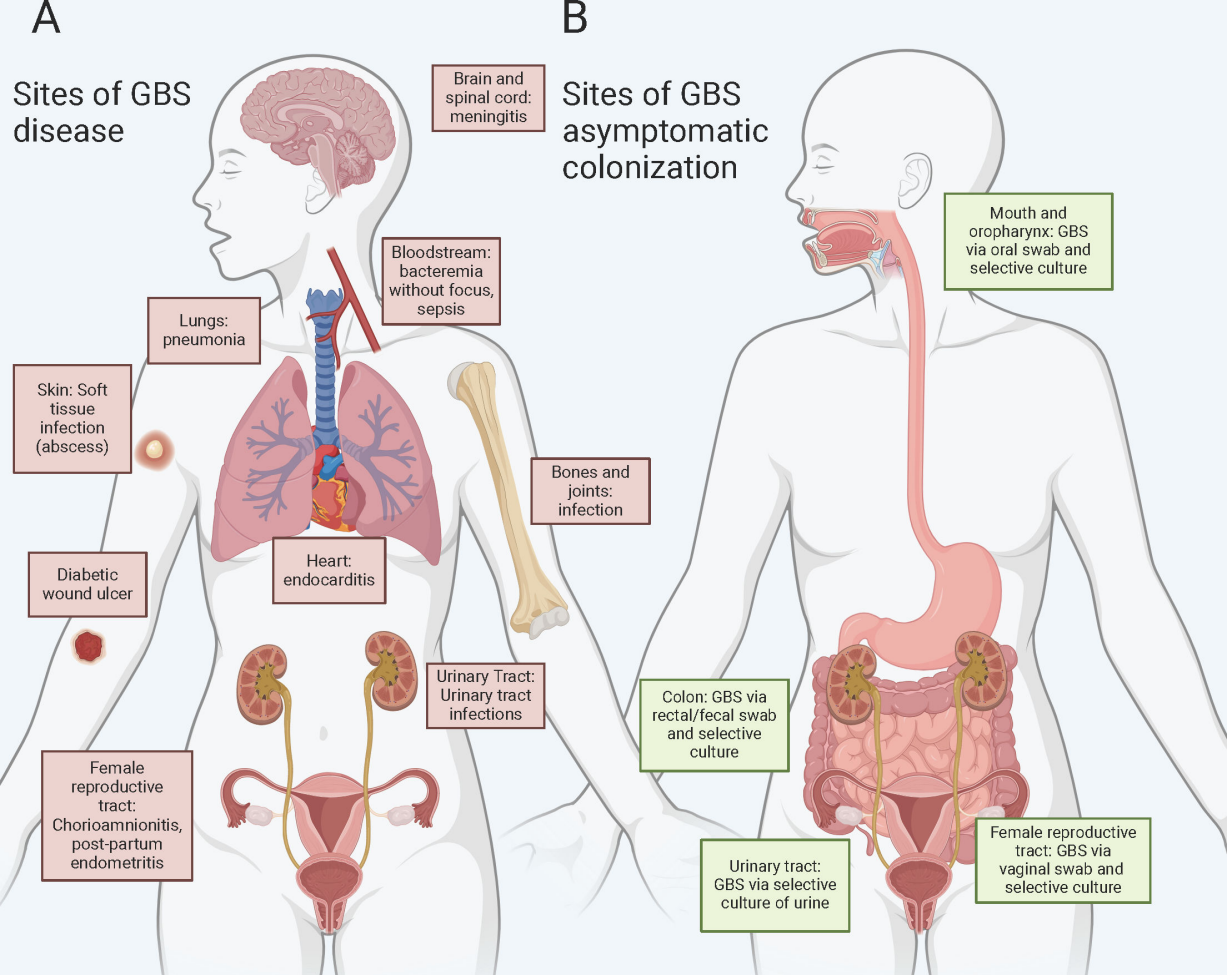
Known body sites of GBS disease and asymptomatic carriage. Summary of the known body sites where (**A**) GBS causes invasive disease (3–11) and (**B**) the known body sites where GBS is an asymptomatic colonizer (12–16). Although GBS has not been cultured from the esophagus, stomach, and small intestine, *Streptococci* are abundant in these niches, as determined by 16S rRNA marker gene analysis (17, 18). Given that (i) GBS has been cultured from the mouth and more distal parts of the GI tract, (ii) GBS has the physiological capabilities to thrive in the environmental conditions in these niches, and (iii) adherence factors capable of interacting with host macromolecules found in these niches, we hypothesize that GBS also resides in these locations. Created with BioRender.com under agreement #RS26EQHEOE.

GBS can cause disease in the form of invasive GBS (iGBS) disease at diverse body sites (Fig. 1A). GBS is most commonly considered to be a pathogen of pregnant people, fetuses, and neonates (2). GBS can asymptomatically colonize the female reproductive tract (FRT) and gastrointestinal (GI) tract and is a common cause of urinary tract infections (UTIs), chorioamnionitis, post-partum endometritis, and bacteremia in pregnant patients, both during pregnancy and postpartum (3). During gestation, GBS can ascend the FRT to colonize the placenta and amniotic fluid resulting in chorioamnionitis (4). Chroioamminoitis is associated with higher chances of stillbirth and preterm birth, which increases the chances of neonatal death (4). In addition, pregnant people who are GBS+ can transmit GBS to the newborn during vaginal delivery, where GBS is the leading cause of neonatal sepsis, pneumonia, and meningitis, causing significant morbidity and mortality worldwide (5).

Two sub-types of iGBS can occur in neonates, early onset GBS disease (EOGBSD) and late onset GBS disease (LOGBSD). EOGBSD occurs within the first week of life and is caused by the vertical transmission of GBS from a GBS+ mother to a neonate before birth or during vaginal delivery. GBS present in the FRT can infect the neonate’s lungs and progress to sepsis and subsequent meningitis (2, 19, 20). Approximately 50% of children born to GBS+ mothers globally become colonized with GBS, and up to 2% of these children develop EOGBSD (5, 21). LOGBSD occurs within the first 7–89 days of life, is more common in children born to GBS+ mothers and preterm infants, and, unlike EOGBSD, is initiated by GBS colonizing the immature neonatal GI tract instead of aspirating into the lungs during delivery (19, 22). While LOGBSD can be caused by vertical transmission during birth, it is also potentially caused by infected breast milk or through horizontal transmission from the community or environmental sources (22–24).

Approximately 34%–46% of all GBS infections reported globally are due to LOGBSD (5). After recovery from EOGBSD or LOGBSD, ~37,000 infants also develop moderate to severe neurodevelopmental impairment globally (5). Taken together, GBS causes ~400,000 symptomatic maternal and infant cases and ~150,000 stillbirths and infant deaths worldwide yearly (25).

In non-pregnant adults and children, GBS presents a significant disease burden by causing urinary tract infections, bacteremia without a focus, pneumonia, sepsis, soft-tissue infections, and bone/joint infections (6, 7). Immunocompromised patients and elderly people are at increased risk for GBS-associated morbidity and mortality (26). Additional underlying health conditions such as diabetes and obesity are suspected risk factors for iGBS (1, 6, 27, 28). GBS is commonly isolated from diabetic wound ulcers and notably absent from non-diabetic wound ulcers (8). Similarly, GBS is also capable of causing urinary tract infections, with prevalence sometimes corresponding to diabetic predisposition (12, 29, 30). While much of what is known about GBS and human disease is in the context of pregnancy, reports from the Active Bacterial Core surveillance network show that the majority of GBS infections occur in non-neonates/non-pregnant people (31). The current overall GBS disease burden in the USA, which is predominantly in non-pregnant people, is likely due to widely implemented antibiotic prophylaxis protocols to reduce transmission of GBS to neonates during birth, which does not impact GBS disease in non-pregnant people (31).

Antibiotics are the primary treatment option for iGBS with penicillin being the first-line antibiotic (second-line antibiotics like erythromycin, clindamycin, and vancomycin are used for those who are allergic to penicillin, although second-line antibiotics are less effective in mitigating EOGBSD than penicillin) (26, 32–34). In many developed countries, pregnant people are prescribed intrapartum antibiotic prophylaxis (IAP) if they are GBS+ or if they have a preexisting risk factor (premature birth, prenatal GBS urinary tract infection, and previous GBS+ delivery) (33, 35). Up to 30% of pregnant people in the USA receive IAP (36). IAP has significantly impacted mother-to-infant GBS transmission during delivery and reduced the incidence of EOGBSD from 1.7/1,000 live births in the 1990s to the current rate of 0.35/1,000 live births in the USA (36). Efficacy estimates for IAP range from 80% to 100% for preventing EOGBSD (37–39). Similarly, penicillin is the first-line antibiotic used for non-pregnant adults with GBS infections. However, IAP has not impacted the incidence of LOGBSD in neonates (40), likely due to LOGBSD arising from GBS that is acquired post-birth (24). Additionally, disease relapse occurs in 4.3% of non-pregnant adults after cessation of antibiotic treatment (41), perhaps due to re-infection with GBS from the skin, the GI tract, or failure of antibiotics to eradicate GBS from deep focal infections. Together, these observations highlight important gaps in our knowledge about disease progression and reservoirs of the GBS that cause LOGBSD in neonates and relapsing infection in adults.

Systematic reviews of global GBS disease burdens highlight the growing threat GBS poses for both pregnant and non-pregnant people (5, 26). New analysis and access to more global data of iGBS burden in pregnant people and neonates suggest that the numbers are higher than previously believed, especially with regard to the number of children who develop neurodevelopmental impairments as a result of iGBS (5). The incidence rate of iGBS in non-pregnant adults globally has also increased over the past few years, especially for older adults with underlying health conditions (26). Unfortunately, GBS is becoming increasingly resistant to antibiotics (42–46). Additionally, it is evident that early-life antibiotic exposure can have long-term health impacts, likely by disrupting key microbiome-immune interactions (47–50). Together, these observations highlight the importance of pursuing alternative strategies to preserve beneficial microbiome-host interactions while mitigating GBS disease.

GBS can cause diverse human infections due to its ability to colonize different body sites. Targeting GBS in one central location such as its GI tract reservoir before it disseminates to other body sites or at-risk individuals could be a more efficient approach to alleviating the GBS burden. Although the adult GI tract is a reservoir for asymptomatic GBS carriage, the physiology of GBS in this environment is understudied. While there are an increasing number of studies focused on GBS neonatal colonization (discussed below), it is unknown what factors (host or microbial) dictate GBS carriage in the adult GI tract. This limits current efforts to develop new, non-antibiotic-based therapeutics and preventative measures against GBS residing in this important niche.

To address this gap, this review synthesizes current knowledge of the common molecular mechanisms GBS utilizes to colonize different body sites. In addition, this review compares physiological similarities between GBS disease sites and the GI tract to hypothesize what common factors GBS may be utilizing in colonizing its GI reservoir. Finally, this review discusses how research focused on GBS in the GI tract will enable more effective and targeted approaches to mitigating GBS disease in a variety of patient populations.

## ASYMPTOMATIC GBS CARRIAGE AT VARIOUS BODY SITES

In addition to being a pathogen, GBS is an asymptomatic colonizer of multiple host-associated niches (Fig. 1B), notably the adult GI and FRT. This is based primarily on data collected from routine antenatal care in the USA and other countries that require universal antepartum GBS screening during the third trimester of pregnancy via vagino-rectal swab followed by selective GBS culture or PCR-based approaches (33, 51, 52). Vagino-rectal swabs of pregnant people demonstrate that 10%–30% of pregnant people are asymptomatically colonized with GBS in their GI and/or FRT (5, 13, 51, 53). In addition, GBS can be detected separately in the urogenital tracts and GI tracts of biological females regardless of pregnancy status (14). Similar asymptomatic carriage rates in non-pregnant adults (independent of biological sex) using selective culture techniques or 16S rRNA marker gene analysis suggest that common risk factors may underlie GBS colonization across diverse human populations (14, 51, 53–57).

Although there is regional variability of GBS colonization rates throughout the globe, behavioral factors like sexual contact/activity and smoking appear to favor GI colonization (53, 55, 58). Additionally, an apparent racial disparity exists with higher rates of GBS colonization in pregnant people seen in racially Black people (53, 55). Regardless, the molecular basis of GBS colonization (and how these may be impacted by various social determinants of health) remains unclear.

Furthermore, it is apparent that the stability of GBS colonization differs between individuals. A longitudinal study of pregnant people from 20 to 37 weeks gestation showed individual changes in both colonization status and GBS serotype over time (59). It remains unclear what biological variables differentiate transient, intermittent, or persistent GBS colonization. However, the regional, intra-, and inter-individual variations in GBS colonization dynamics suggest directions for future, more targeted studies to provide mechanistic insights. Furthermore, the stratification of patients based on the stability of GBS colonization suggests that it is highly responsive to dynamic host-, microbiome-, or environmentally determined variables.

So far, surveys of GBS carriage rates and associated risk factors have focused on rectovaginal carriage, which likely obscures niche-specific differences between the two body systems and hinders the development of targeted strategies for decolonizing GBS carriers. Future studies focused on asymptomatic GBS in the GI tract will enable a more robust understanding of the variables impacting carriage in this key niche across all populations. Longitudinal or cross-sectional human studies that account for variables like microbiome composition/microbiome-produced metabolites, immune status, health history, and lifestyle parameters (e.g., diet, environmental exposures, and socioeconomic status) could help to identify specific variables that dictate GBS carriage. Additional studies using rodent models of GBS GI colonization could provide a highly controlled foundation to understand the impacts of variables like diet, inflammation, and the microbiome, as in our previous and ongoing work with the diarrheal pathogen *Clostridioides difficile* (60–62).

## OUR LIMITED UNDERSTANDING OF GBS GI COLONIZATION

From the GI reservoir, GBS can transmit to other body sites (e.g., the urogenital tract) and to others at risk for GBS disease (e.g., neonates) (2, 5, 14). These observations situate the GI tract as an important niche for GBS and suggest that targeted interventions to de-colonize GBS GI carriers would be effective in decreasing GBS transmission to other body sites and others at risk for disease. However, such approaches are not currently possible due to a relatively limited understanding of the host-, microbiome-, and GBS-driven parameters that dictate GBS GI carriage.

First, most work done to determine the rates of GBS carriage in the GI tract relied on rectal swabbing and fecal sampling. Therefore, GBS colonization of the proximal regions of GI tract is understudied. This limits our understanding of how these body sites could serve as reservoirs for GBS that subsequently translocate to the distal GI and other body sites. Despite these gaps in the GBS literature and although GBS has not been directly cultured from many locations in the proximal GI, it is evident that *Streptococci* are prevalent and abundant inhabitants of the upper GI tract, spanning from the mouth (15, 63), oropharynx/esophagus (16, 64, 65), stomach (17, 66, 67), and small intestine (18, 68, 69). *Streptococci* are less common in the distal GI tract than in the proximal GI tract, as observed through 16S rRNA marker gene analysis (17, 70). This biogeography differs substantially during diverse conditions, including pregnancy, where elevated *Streptococci* in the distal gut are a prominent signature of a disrupted, “dysbiotic,” microbiome (71–73). While not a dominant member of the proximal GI based on 16S data, GBS is directly detected by culturing and multi-locus sequence typing in saliva (15) and oropharyngeal swabs from healthy humans (16, 65). This suggests previous 16S-based assays of samples collected from other locations in the proximal GI may not have adequately captured the prevalence and abundance of GBS. Furthermore, because 16S-based assays do not account for non-bacterial members of microbiomes (e.g., fungi and bacteriophages), important trans-kingdom relationships may have been previously overlooked. Determining how GBS interacts with other *Streptococci* and unrelated microbiome members in the proximal GI will provide a foundation for how the proximal GI could seed the distal GI, other body sites (e.g., the FRT), and others at risk for infection (e.g. neonates).

While numerous microbiome members are implicated to have synergistic or antagonistic relationships with GBS across body sites in health and diseases, existing studies of these interactions in the GI tract are limited and predominantly association based (74). For example, GBS presence in infant stools is positively associated with *Lactobacillus* and *Staphylococcus* species, but it is unclear if these associations are generalizable to more complex adult microbiomes (75). Rectal-vaginal swabbing-based studies showed GBS co-occurs with *Staphylococcus aureus and Candida albicans*; however, it is unclear whether these associations are present in the rectum, vagina, both, or neither (e.g., it is possible that vaginal *C. albicans* is associated with rectal GBS) (76–80). Finally, other studies that used vaginal swabs showed co-occurrence between GBS and common gut commensals such as *Escherichia coli* (14, 81), *Akkermansia* spp. (82), and *Prevotella* spp. (83, 84), and although these associations were seen in the vagina, it is possible that they are also relevant in the GI tract. Therefore, it is evident that a more focused study of GBS-microbiome interactions is needed.

## BORROWING PARADIGMS FROM EXISTING EXPERIMENTAL SYSTEMS TO INFORM FUTURE STUDY OF GBS IN THE GI TRACT

A wide variety of animal models have been developed to study GBS. Murine models of adult vaginal colonization, adult oropharyngeal colonization, and infant gut colonization (which predisposes LOGBSD) recapitulate many relevant aspects of human colonization/disease (85–87). The murine model of neonatal GBS meningitis acquired through vertical transmission from vaginally colonized pregnant female mice replicates what is seen in humans, where learning and memory are impaired in surviving newborns that reach adulthood (88). Rat models of vaginal colonization have been used to study vaginal vertical transmission of GBS (89). Non-human primate models enable the study of *in utero* transmission (similar to ascending GBS infection) of GBS from mother to infant via intraamniotic or choriodecidual inoculation (90–95). While these model systems, combined with powerful *in vitro* experimental tools [e.g., targeted mutagenesis (96), multi-omics (97, 98), fluorescent imaging (99), and biofilm assays (100)], have provided key insights into the molecular and genetic basis for GBS colonization and pathogenesis in a variety of host-associated ecosystems, no comparable models have been developed to study GBS in the adult GI tract. Given the emerging view of the GI tract as a reservoir for GBS, this lack of experimental tools limits our understanding of GBS biology and possible improved therapeutic options.

To inform possible foundations and directions for the GI-centric study of GBS, we focus on four important themes from the existing bacterial pathogenesis and microbiome literature. First, it is evident that GBS adherence to surfaces, host cells, and bacterial cells is important throughout the body and that similar adherence mechanisms may promote colonization of the GI tract. Second, chemical and physical similarities exist between various body sites and the GI tract. These similarities may help inform research directions aimed at targeting aspects of GBS physiology or virulence that are dependent on these environmental characteristics. Third, host-determined variables like diet are powerful tools to alter the composition of microbial communities within the GI tract, which could be leveraged to disfavor GBS colonization. Fourth, given that other pathogenic bacteria colonize the GI tract and cause disease at other body sites (i.e., are gut-resident pathobionts), we can draw from similar efforts aimed at targeting other pathogenic bacteria in their GI reservoirs.

### Adherence to host cells, surfaces, and other microbiome members

GBS colonizes and causes disease throughout the human body due in part to its versatile surface-associated macromolecules that enable adherence to host cells and host extracellular matrix (ECM) components (fibrinogen, fibronectin, and laminin) (101, 102). Different proteins target different ECM components or host cell features, most of which are shared between cell types and body sites, including the GI tract (Table 1). Below, we discuss adherence factors encoded by diverse GBS isolates, their known impacts on GBS colonization/disease, and their possible roles in the GI tract. Importantly, these adherence factors are not universal to all GBS isolates, and this strain-to-strain variability should be considered when comparing studies that used different GBS strains.

**TABLE 1:** GBS-encoded factors involved in adherence to surfaces^*a*^

GBS-encoded factor	Interacts with host factor, surface, or microbial components	Relevant body sites/cell types (confirmed *in vivo* and hypothesized based on *in vitro* studies)
Srr1 and Srr2 (surface protein)	Binds to host fibrinogen (103)	Vagina (104, 105) Astrocytes and neurons (106) (Central nervous system: meningitis) human brain microvascular endothelial cells (HBMECs) (107) infective endocarditis (108)
Lipoteichoic acid (LTA, surface)	Anchor to host cells (106, 109)	HBMECs (106, 109)
Pili islands: PI-2a PI-2b	Anchors to host cells and possible biofilm formation (99, 110–112); binds to epithelial and endothelial cells (110); increased survival after macrophage engulfment (112); adherence to MUC5B (99)	Biofilm formation (111) HBMEC and lung epithelial cell line (110) Vagina (99)
FbsA	Binds fibrinogen (113)	Adherence to human epithelial cells (9) Mediates platelet aggregation (114) HBMECs adherence (115)
FbsB	Binds fibrinogen (10)	Promotes host cell invasion (10)
FbsC	Binds fibrinogen (11)	Promote virulence, adherence, and invasion of epithelial and endothelial cells and form biofilms (11) Vagina epithelial cell binding *in vitro* (116)
SfbA	Binds fibronectin (117)	Invasion of brain endothelium causing meningitis (117) Vaginal and cervical epithelial adherence (117)
ScpB	Binds fibronectin (118)	Adherence to lung epithelial cells (118)
Lmb	Binds to laminin (119)	Enable HBMCE invasion (120)
BibA	Binds epithelial cells (121)	Adherence to lung and cervical epithelial cells (121)
Capsular polysaccharide	Anchors to host cells (sialic acid interacts with host cell glycans) (122)	Biofilm formation (123) Promotes virulence and colonization of rectal and vaginal epithelium (123, 124)

^*a*^ Summary of the different surface-associated macromolecules GBS uses to bind to host cells and/or ECM components, promoting colonization and virulence.

Fibrinogen-binding proteins (FbsA, FbsB, and FbsC) and serine-rich repeat glycoproteins (Srr1 and Srr2) enable both GBS colonization and virulence. FbsA increases adherence of GBS to human epithelial cells (9), and FbsB is vital for GBS invasion of host tissue, specifically lung epithelial cells (10). In addition to promoting GBS adherence and invasion of human cervical epithelial and human brain microvascular endothelial cells (HBMECs), FbsC promotes GBS biofilm formation *in vitro* (11, 116). Srr1 and Srr2 proteins bind to fibrinogen Aα chains found in the vagina, promoting adherence and colonization *in vivo* (103–105, 125). FbsA and Srr1 are also crucial to GBS-mediated meningitis, perhaps due to HBMECs, brain astrocytes, and neurons constitutively expressing these fibrinogen chains (107, 115, 126). FbsA and Srr1 also bind platelets, an important first step in GBS-induced thrombosis and infective endocarditis (108, 114). Fibrinogen is constitutively expressed by human intestinal epithelial cells as its byproduct, fibrin, is used in epithelial cell wound healing (127), suggesting that these proteins may be important for GI colonization.

Fibronectin-binding proteins, such as SfbA and GBS C5a peptidase (ScpB), mediate GBS adhesion to host epithelial cells by binding fibronectin found in the ECM (117, 118). Additionally, fibronectin is found throughout the ECM of intestinal epithelial cells and is a common target for bacterial adhesion by other organisms in the GI, such as *Lactobacillus* spp., and other *Streptococci,* such as *Streptococcus pyogenes* (128–130).

Laminin makes up the basement membrane of the ECM below the epithelial and endothelial layers, acting as a physical barrier preventing bacteria from disseminating (131). Laminin-binding protein (Lmb) allows GBS to colonize damaged epithelial cells and favors subsequent translocation into the bloodstream (119). Lmb also supports the GBS invasion of HBMECs (120). Notably, the lamina propria is a thin layer of connective tissue beneath the epithelium-containing laminin (132). Adherence to this layer in the context of epithelial damage could benefit GBS-persistent colonization of the GI and potential dissemination to other body sites. In addition to being able to adhere to ECM components, GBS also expresses metallopeptidases capable of cleaving them, potentially enabling GBS to invade the host tissue and disseminate (133).

Another GBS-encoded adhesin is immunogenic bacterial adhesin (BibA) that is involved in GBS adherence to lung and cervical epithelial cells (121). In addition, GBS vaginal adherence protein (BvaP) is a secreted and cell-surface-associated protein conserved across GBS strains important for GBS adherence to ECM components and human vaginal epithelial cells (134). However, the interacting host components for BibA and BvaP are not known, limiting predictions of whether they would be relevant in the GI tract.

GBS strains are typically characterized by serotyping analysis based on the expression of 10 distinct type-specific capsular polysaccharide antigens (Ia, Ib, II, III, IV, V, VI, VII, VIII, and IX) (135, 136). The capsular polysaccharide side chains have terminal sialic acids that enable adherence to host cells (122). Capsule expression increases biofilm formation and promotes strains ability to colonize in the FRT via epithelial cell anchoring as seen in a murine model (123). GBS adherence to human rectal and vaginal epithelial cells *in vitro* differs based on GBS serotype (124). Additionally, different serotypes show differences in virulence and prevalence in humans, suggesting that differences in capsules impact GBS colonization ability (25). Serotypes Ia, III, and V are the most commonly isolated serotypes that cause disease in humans (25–27, 137, 138). In particular, for neonatal disease, Serotype III causes 48% of EOGBSD and 74% of LOGBSD globally (25). In non-pregnant adults, Serotype V is the most prevalent serotype globally, followed by Ia and III, which vary in prevalence at different study sites (26). Capsule-dependent fitness is evident in a LOGBSD mouse model of GI colonization, which showed that a serotype Ia strain outcompetes a III strain (139).

GBS strains also utilize different types of pili (PI-1, PI-2a, and PI-2b) to aid in adhesion to host cells and possible biofilm formation (104, 140). It is possible for strains to encode a combination of up to two pili on genetically distinct pilus islands. The pili distribution and possible combinations in iGBS from clinical isolates vary regionally with PI-2a (80%–90%) being the most prevalent followed by PI-1 (70%–86%) and PI-2b (7%–21%). The most prevalent combination is PI-1 with PI-2a (110, 140–143). This distribution is important in understanding the range of GBS adhesion phenotypes. Most notably, only GBS strains with PI-2a are capable of biofilm formation, a valuable ability that enables long-term persistence and colonization of GBS on surfaces, while PI-1 does not play a role in cell adherence to host epithelial cells (111, 144). PI-2b enables increased GBS adherence and invasion of epithelial and endothelial cells (110, 112).

GBS uses pili to colonize the FRT. The vaginal epithelium benefits from the mucins produced by the cervix, which help protect against infection by preventing bacterial adhesion and capturing potential pathogens within mucus aggregates (145). MUC5B, the predominant gel-forming mucin found in the vagina, inhibits GBS adhesion to human vaginal epithelial cells and prevents GBS from ascending upward to the cervix in an *in vivo* murine model. In response to MUC5B, GBS conversely upregulates PI-2b to increase its adherence and persistence in the vagina (99). While this is the first identified direct interaction between a GBS factor and a mucin glycoprotein, other *Streptococci* can interact with other mucin types to establish colonization in the oral cavity and upper GI. For example, *Streptococcus gordonii* is able to bind the salivary mucin MUC7 to colonize the mouth (146).

MUC5B is also found on other mucosal surfaces, including the oral cavity (salivary glands) and GI tract (stomach and colon) (147–149). This suggests that GBS may utilize pilus islands to maintain colonization in the GI tract like how it does in the vagina. The GI tract produces a multitude of other mucins, most prominently MUC2, and all these mucins may have differing negative or positive effects on GBS adherence in the GI tract.

### Similar chemical and physical characteristics between the GI tract and other host-associated GBS niches

Gradients of pH, oxygen availability, microbial diversity and density, and nutrient availability create diverse environments through the longitude of the GI tract (69, 150). Many of these environments share features with other sites of GBS carriage and disease (Table 2) and provide a framework for understanding possible fitness determinants of GBS GI colonization.

**TABLE 2:** Sites of GBS disease and asymptomatic colonization^*a*^

Body site	pH	Oxygen availability	Microbial biodiversity/biomass	Predominant mucin types	GBS presence
GI Tract:					
Mouth	6.7–7.3 (151)	Aerobic/atmospheric (~145 mmHg and ~21% O2) (152)	~740 bacterial species (153, 154) 10^4^–10^5^ CFU/mL in human saliva (155) ~10^10^ CFU total (156)	MUC5B, MUC7, MUC19, MUC1, and MUC4 (157)	GBS presence detected via selective culture (15)
Esophagus	7(158)	Aerobic/atmospheric (~145 mmHg and ~21% O2) (152)	95 species-level operational taxonomic units (OUTs) (159) 10^4^ CFU/mm^2^ of mucosal surface (159)	MUC5B, MUC1, and MUC4 (160)	GBS not detected; Streptococci dominant genus via 16s sequencing (17)
Stomach	2–3.5 (lumen) (161) 4.6–7 (mucus layer) (161)	Lumen: 25–58 mmHg (162, 163)	10^1^–10^3^ CFU/mL in humans (164)~100 OUTs (17, 66)	MUC5AC, MUC6, MUC2, and MUC1 (165, 166)	GBS not detected; Streptococci dominant genus via 16s sequencing (17)
Duodenum	6 (167)	32–60 mmHg (150, 163)	~380 genera (168)10^1^–10^3^ CFU/mL in humans (164)	MUC2 and MUC6 (147)	GBS not detected; Streptococci dominant genus via 16s sequencing (18)
Jejunum	7–7.5 (167)	Undetermined	>220 species (169, 170)10^4^–10^7^ CFU/mL in humans (164)	MUC2 and MUC6 (147)	GBS not detected; Streptococci dominant genus via 16s sequencing (18)
Ileum	7.5 (167)	Lumen: ~10 mmHg (2%) (150) Crypt-lumen interface: 11 mmHg (2%) (171)	280 OTUs and 229 identified species (172)10^4^–10^7^ CFU/mL in humans (164)	MUC2 and MUC6(147)	GBS not detected; Streptococci dominant genus via 16s sequencing (18)
Cecum	6.3 (164)	Lumen: 1 mmHg (150)	>1,000 species (173)10^11^–10^12^ CFU/mL in humans (164)	MUC2 (147)	GBS detected via selective culture (14)
Colon	6.5–7 (167)	Lumen: 3 mmHg (~0.4%) (171)	>1,000 species (173)10^11^–10^12^ CFU/mL in humans (164)	MUC2 (147)	GBS detected via selective culture (14)
Vagina/FRT	3.8–4.5(174)	15–35 mmHg (175)	10^7^–10^8^ CFU/mL (176, 177) 300 + bacterial species (178, 179)	MUC5B, MUC5AC, MUC6, MUC4, and MUC1 (180)	GBS detected via selective culture (3)
Cerebrospinal fluid	7.33 (181)	Basilar cisterns: 65 mmHg (182) Third ventricle: 130 mmHg (182)	NA	NA	GBS detected via selective culture (5)
Urinary tract	Urine: 5–6 (183)	Bladder: 23–45 mmHg (184)	~562 species (185) <10^5^ CFU/mL (186)	Kidney: MUC1, MUC12, MUC13, and MUC20 (187)	GBS detected via selective culture (29)
Bloodstream	7.35–7.45 (188)	Dissolved oxygen in plasma: 0.3 mmHg or 0.3% (189) Oxygen bound to RBCs: 100 mmHg (189)	NA	NA	GBS detected via selective culture (19)

^*a*^ Summary of relevant physical, chemical, and microbial characteristics of body sites where GBS colonizes asymptomatically or causes disease. Hypothesized sites of GBS colonization (see Fig. 1 legend) are also described.

Notably, the vagina is an acidic (pH: 3.8–4.5) and anaerobic (15–35 mmHg O_2_) environment with low microbial biodiversity relative to other host-associated niches like the distal GI tract (174, 175, 178). In the GI tract, the stomach and proximal small intestine are also acidic environments with low microbial diversity (161). While these regions are more aerobic than the vagina, the large intestine has similar oxygen levels to the vagina (150). Parallels could be drawn between how GBS overcomes these physical barriers in the vagina to understand how it is able to colonize in the GI tract.

In addition, while the blood-brain barrier (BBB) is regarded as an impermeable layer of endothelial cells held together by tight junctions (190), it must still allow the passage of necessary nutrients into the central nervous system (CNS) while preventing microbial invasion. GBS bypasses the physical defenses of the BBB after adhering to endothelial cell surfaces (109, 191). On the other hand, the GI tract consists of a single layer of epithelial cells held together by tight junctions and selectively enables the absorption of nutrients while excluding bacteria from passing through (192). While it is not yet known if and how GBS is able to translocate through the GI epithelium, possible pathways GBS exploits could be gleaned from our current understanding of how it crosses the BBB.

Upon crossing the BBB, GBS must survive in the cerebrospinal fluid of the CNS, a neutral and well-oxygenated environment (181, 182). Cerebrospinal fluid has common features with the colon (pH) and with the upper GI (oxygen levels) (152, 167). Therefore, existing knowledge of GBS physiology in this environment and in response to these important physiological parameters may counterintuitively be helpful for understanding its colonization of various GI niches with neutral pH or high oxygen levels.

### Differences in microbiome composition/function to dictate GBS carriage

GBS is a common bacterial species isolated from diabetic wound ulcers but is rarely isolated from non-diabetic wound ulcers (8). In diabetic wound ulcers, GBS *cyl* operon is upregulated, leading to noticeably more hemolysin/pigment production compared to GBS isolated from non-diabetic wounds (193). Additionally, CylE has been shown to be important in GBS murine models of sepsis (194). In diabetic mice, GBS can overcome the nutritional immunity of host chelation of zinc, manganese, and nickel to persist in a diabetic wound (195). In addition, the adaptive metabolism of GBS enables its survival in the hyperglycemic environment of a diabetic wound (8). While GBS mainly utilizes fermentation for energy production, if it is able to scavenge heme and quinones from its environment or other bacteria present, it is able to perform cellular respiration, thereby increasing virulence *in vivo* (196, 197). This suggests GBS fitness can be enhanced by other microbes, raising the question of what other body sites GBS is able to colonize in part due to microbe-microbe interactions and whether phylogenetically/functionally similar organisms play similar roles at different body sites (Table 2). Additionally, the heightened fitness of GBS in an environment high in glucose concentration suggests it may also thrive in an environment with higher levels of free simple sugars, like the small intestine. These types of interactions can be readily studied by developing animal models of GBS GI colonization and by using tools like dietary manipulation, which rapidly and reproducibly alter the composition and metabolic output of GI-resident microbiomes (60, 198).

### Common themes between GBS and other pathogens

Some GI commensals can switch to a pathogenic lifestyle depending on changes to the GI environment, translocation to other parts of the body, or transmission to at-risk individuals. These organisms are referred to as opportunistic pathogens or “pathobionts” (199). For example, *Enterococcus faecalis* is a gut commensal capable of translocating to the bloodstream, causing sepsis and liver abscesses during dysbiosis and subsequent *E. faecalis* overgrowth (i.e., after antibiotic treatment) (200, 201). Excitingly, inhibition of *E. faecalis* from moving out of its GI reservoir can prevent systemic infection in mice (202). Uropathogenic *E. coli* normally originates from the GI tract and subsequently colonizes the urinary tract to cause UTIs. Recurrent UTIs can occur in part due to reinoculation from strains residing in the GI tract (203). Other commensal *Streptococcal* species such as *Streptococcus mitis* are also pathobionts, causing infective endocarditis and bacteremia outside of their oropharyngeal reservoir (204). Finally, *Pseudomonas aeruginosa* is a notable example of an organism that can cause infection in different body sites (notably the bloodstream and lungs) upon spreading from its GI niche (205, 206). Given that GBS is a resident of the adult GI and can be pathogenic at other body sites or be transmitted to others at risk for infection, it is evident that it is a gut-resident pathobiont. Principles learned from other gut-resident pathobionts can inform research on GBS in the GI tract and possible convergently evolved strategies of switching between commensal and pathogenic lifestyles. Conversely, advances in understanding the gut-resident niche of GBS could inform our understanding of other phylogenetically diverse pathobionts.

## FUTURE PROSPECTS FOR THERAPEUTICS ENABLED BY A BETTER UNDERSTANDING OF GI COLONIZATION

The status quo for preventing and treating GBS infections in humans across the lifespan is antibiotic use. Though GBS strains resistant to penicillin have not been reported, GBS isolates with increased minimum inhibitory concentrations to penicillin have been reported in Japan and North America due to mutations in penicillin-binding proteins (42–44). In addition, resistance is rapidly emerging to second-line antibiotics (e.g., clindamycin) used for people with penicillin allergies (45, 46).

**Fig 2:**
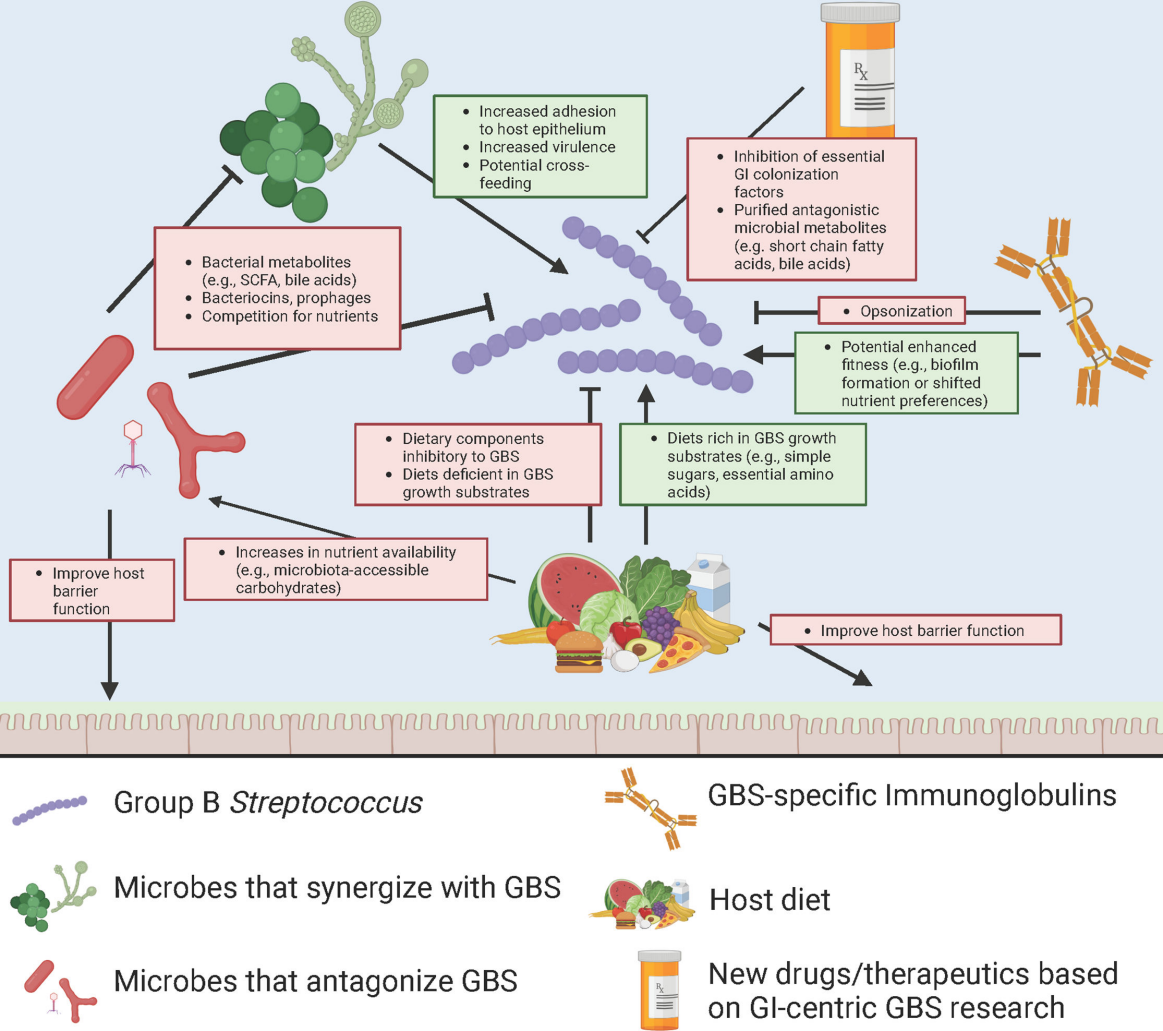
Prospects for therapeutics enabled by a better understanding of GI colonization. Summary of potential GBS-microbiome and GBS-host interactions that could be targeted with a better understanding of GBS in the GI tract. Created with BioRender.com under agreement #IEN263N1AK5.

IAP is also associated with short-term negative health impacts on mothers and neonates and possible long-term side effects on neonates. For example, Gram-negative bacterial infections are more common in neonates whose mothers received IAP, and there is an increased risk of post-partum infections in mothers who received IAP (e.g., *C. difficile* infection) due to impacts on maternal microbiomes (207, 208). IAP also alters the neonatal microbiome, with significant impacts on culturable bacteria from infant feces, α/β diversity metrics, and decreased transmission of beneficial microbes like *Lactobacillus* from the mother (209–211). Emerging evidence from multiple large prospective cohort studies shows that early life antibiotic exposure increases the likelihood of a variety of adverse health outcomes in children (e.g., allergic rhinitis, atopic dermatitis, asthma, reduced growth, and enhanced high-fat diet-induced obesity) (48–50). These observations are part of a growing body of literature that highlights the deleterious off-target effects that antibiotics have on beneficial microbes and how this can impact diseases later in life (47), all while contributing to growing antibiotic resistance. To improve health outcomes from GBS-associated diseases, new and precise approaches to mitigate GBS infections are needed, and a better understanding of GBS in the GI tract could enable new therapeutics to be developed more effectively (Fig. 2).

### Vaccination

One possible alternative to antibiotics is to administer anti-GBS vaccines to pregnant people and other at-risk populations. Vaccine candidates include multi-valent glycoconjugate vaccines to immunize against GBS capsular polysaccharides and protein-based vaccines to immunize against surface-exposed proteins expressed by multiple serotypes (212). Observations of naturally acquired humoral immunity to GBS help establish the rationale for vaccination. High concentrations of maternal anti-capsular polysaccharide-specific IgG in serum are associated with reduced homotypic GBS colonization and a reduced risk of EOGBSD in newborns (59, 213). In addition, data from phase I/II clinical trials of anti-GBS vaccines support the efficacy of maternal vaccination. For example, maternal vaccination with glycoconjugate vaccines increases capsule-specific IgG levels in infants for approximately 3 months post-delivery (214, 215). Current maternal vaccination strategies focus on targeting GBS capsular polysaccharide in a multivalent manner to increase protection coverage (216). Thus, two possible synergistic effects from maternal vaccination would reduce GBS disease in neonates—passive immunity to GBS in newborns and reduced GBS burdens in pregnant people. Another trial similarly showed elevated IgG in non-pregnant adults for 6 months post-vaccination (212), providing further rationale for the continued development of anti-GBS vaccines to benefit humans across the lifespan.

However, these studies have primarily focused on IgG, the most dominant Ig isotype in serum. Due to GBS being prevalent in the gut, it would be beneficial to understand how antibodies in the GI tract, especially IgA (the dominant isotype in the gut), impact GBS GI colonization. Though IgA coating of bacteria is widely accepted to have negative consequences on bacterial fitness, two previous studies using *E. coli* highlight that IgA can enhance bacterial fitness. In the first of these studies, *in vitro* assays using type 1 pili-expressing *E. coli* showed that IgA enhanced biofilm formation (217). Work with other phylogenetically diverse bacterial species has shown that biofilm formation enhances bacterial fitness in the gut using animal models (218). An additional study that used dimeric monoclonal IgAs (mIgAs) in gnotobiotic mice showed that mIgAs exerted antigen-specific effects on *E. coli* fitness, like reduced susceptibility to bacteriophage infection, reduced bile acid sensitivity, altered nutrient uptake, reduced motility, or increased aggregation (219). Though these previous studies are a powerful demonstration of the pleiotropic effects of IgA on a specific bacterium, it is not immediately clear how these findings translate to other bacteria or how they impact microbes within conventionally reared mice or humans. Regardless, these data indicate that IgA can have diverse positive and negative impacts on bacterial fitness in the gut.

Given the importance of GBS biofilm formation in colonization, understanding if and how IgA enhances biofilms *in vivo* is vital to determining possible off-target effects of current vaccination efforts (111). It is possible that IgA could either positively or negatively impact GBS fitness, and these effects may be distinct from the specific antigen(s) targeted (e.g., pili, capsular polysaccharide, or other surface-exposed antigens). Notably, proteins that bind the Fc region of human IgA have been characterized in some GBS strains, suggesting that GBS evolved ways to protect against the antagonistic effects (or enhance the positive effects) of IgA binding (220).

Despite the promising results from phase I/II clinical vaccine trials and observational studies on naturally acquired humoral immunity to GBS, there are concerns of maternal vaccine hesitancy and serotype replacement (as seen after vaccination with multivalent glycoconjugate vaccines for *Streptococcus pneumoniae*) (221, 222). Additionally, to maximize vaccine safety for pregnant people, GBS vaccinations are given during the third trimester, which limits the ability to provide a multi-shot regimen. This puts pressure on a single vaccination to promote a sufficient antibody response (223). This could be engendered by utilizing self-assembling virus-like particles conjugated to GBS capsular polysaccharides (224). Taken together, future vaccination work should consider the ways IgA impacts GBS in the gut to enable a more efficient and desired vaccination response. This work could begin by identifying whether GBS-specific IgA correlates with GBS burdens/disease severity and which epitopes are predominantly targeted by IgA. Subsequently, the effects of IgA on targeting specific epitopes (e.g., capsule, pili, and other GBS surface exposed epitopes) could be defined using monoclonal antibodies in order to prioritize vaccine targets.

### Probiotics/dietary intervention

Meta-analysis of synergistic and antagonistic interactions between GBS and other GI-resident microbes suggests possible interactions that can be leveraged to discourage GBS colonization (74). To this extent, a deeper understanding of the molecular basis for these interactions will positively impact efforts to target GI-resident GBS. Similar microbiome-based approaches have been applied to recurrent *C. difficile* infections (rCDI) with fecal microbiota transplantation (FMT). FMTs are increasingly common procedures aimed at adjusting the patient’s microbiome back to a “healthy” state in order to resolve rCDI (225). More controlled approaches, based on rationally designed microbial consortia to decrease *C. difficile* infection severity, first showed therapeutic potential in murine model CDI (226). More recently, a phase III, double-blind, randomized, placebo-controlled trial of an orally delivered probiotic consisting of Bacillota spores showed a decrease in rCDI in humans (227). Beyond *C. difficile*, a separate phase II, double-blind, randomized, placebo-controlled trial showed that *S. aureus* carriage in the GI tract could be reduced using a probiotic preparation of *Bacillus subtilis* spores (228). *B. subtilis* was previously shown to inhibit *S. aureus* quorum-sensing *in vitro* and in a murine model (229). This work with *C. difficile* and *S. aureus* highlights the utility of understanding microbe-microbe interactions using controlled experimental approaches to enable effective probiotics.

Several clinical trials used commercially available probiotics to reduce asymptomatic GBS carriage in pregnant people, with the goal of decreasing vertical transmission of GBS to neonates. However, these efforts have had mixed success. A meta-analysis of six clinical trials showed that probiotics were inconsistently effective in decreasing GBS burdens in pregnant people. Specifically, some trials showed a statistically discernible decrease in GBS count and GI symptoms, while others saw no statistical difference (230). Similarly, a phase II, randomized controlled trial of an oral probiotic shown previously *in vitro* to limit GBS fitness showed a slight difference in GBS colonization (5% decrease) in humans but was not statistically discernable (*P* = 0.73) in part due to being underpowered (231, 232). Another oral probiotic consisting of *Lactobacillus reuteri* decreased the rate of mother-to-child transmission of GBS [6% in the probiotic group and 22% in the control group (*P* = 0.09)] (233). Taken together, these observations demonstrate that probiotics are variably successful in mitigating mother-to-infant GBS transmission. Given that rationally designed probiotics show promise for mitigating other bacterial infections [e.g., *C. difficile* and *S. aureus* infections (227, 229)], a better understanding of how other microbes affect the fitness of GBS will enable a more tailored probiotic approach.

Diet is another possible way to leverage the host microbiome to target GBS GI colonization. For example, in humans, host fiber consumption influences the fitness of other pathogens, such as *C. difficile* and *Shigella* (234–236). Short-chain fatty acids are also notable end-products of bacterial fiber metabolism that have an impact on *C. difficile* abundance in the mouse GI tract (237). Therefore, future research on how various dietary components and the resulting microbial metabolic end-products influence the GBS colonization tract may directly identify diet-based strategies or indirectly identify microbiota members and metabolites relevant for reducing GBS GI carriage in humans. This work could begin by using controlled dietary conditions or probiotic supplementation in animal models of GBS colonization/disease to identify dietary components and altered microbial/metabolic signatures that correlate with GBS burdens/disease severity. Subsequently, direct interactions could be investigated with molecular/genetic approaches to inform interventions in at-risk human populations.

### Promoting gut barrier function

As IAP has shown no impact on LOGBSD (40), a better understanding of how GBS colonizes the neonatal gut is likely needed to develop therapeutics for this disease type. Work in LOGBSD mouse models showed that GBS toxin induces transcriptome changes in the host colon, specifically toward genes involved in epithelial barrier integrity and immune signaling (22), and the capsule is important for GI colonization (139). Additionally, recent work has shown that GI immaturity due to premature birth is a risk factor for neonate colonization of GBS and subsequent meningitis (23). Therefore, one approach to LOGBSD could be focused on promoting gut barrier function. This could occur through diet-based strategies [e.g., human milk oligosaccharides directly enhance gut barrier function (238)] or through probiotic intervention [e.g., *Bifidobacterium longum* subsp. *infantis* EVC001 improves barrier function in infants (239)]. Notably, *Bifidobacterium* spp. are negatively associated with GBS carriage in neonates with some strains showing antimicrobial properties against GBS (209). Understanding these *Bifidobacterium-*GBS interactions (or possibly other strains with greater anti-GBS activity) in the GI would enable a more effective probiotic that can both promote gut barrier function and decrease GBS colonization in adults and LOGBSD in neonates. To begin to identify dietary components or microbial metabolites that improve barrier function, purified metabolites or metabolite extracts from prominent gut microbiome members could be applied to monolayers of Caco-2 cells, and improvements in transepithelial electrical resistance could be measured. As needed, fractionation techniques/small molecule discovery pipelines could be used to prioritize metabolites with therapeutic potential. To begin to identify specific antagonistic molecular interactions between GBS and other microbes, a variety of co-culture and *in vitro* GBS-killing assays could be used to identify organisms that antagonize GBS. Subsequently, direct interactions could be investigated with molecular/genetic approaches to inform interventions in at-risk human populations.

## CONCLUSIONS AND FUTURE DIRECTIONS

GBS presents a real and growing threat to humans as current antibiotic treatment options become less effective. Additional unintended negative effects of antibiotics, such as early-life microbiota disruption, highlight the need for new approaches. One possible approach involves selectively depleting GBS in its GI commensal niche to discourage colonization at other body sites and transmission to at-risk individuals (e.g., the FRT and neonates, respectively).

In addition to infecting humans, GBS is a leading cause of bovine mastitis (240), highlighting that a better understanding of GBS and improved efforts to mitigate GBS disease are aligned with the CDC’s One Health initiative, which seeks to achieve optimal health outcomes by recognizing the interconnection between people, animals, plants, and their shared environment. Therefore, the successful development of new prevention and treatment approaches to combat GBS is likely to have broad impacts on public health and agriculture.

Work done with other GI pathobionts highlights the feasibility of a reservoir-focused approach to treatment. GBS is considered a GI commensal, but not much is known about its biogeography in the gut or the molecular tools it utilizes to colonize. What is better understood is how GBS colonizes the body sites where it causes disease, including the FRT and BBB. We identify common themes shared by these varied body sites. Based on these similarities, we highlight possible molecular factors GBS uses to colonize the GI tract. Additionally, we highlight how the varied sites of GBS colonization and disease also have physiological similarities to the host GI tract, underlining how current knowledge of GBS colonization and disease can be used as a foundation to understand GBS in the GI tract. Finally, we highlight how a GI tract- and intestinal microbiome-focused approach to targeting GBS can enable new and impactful therapeutics while minimizing possible collateral damage to our microbiomes.
